# Risk factors and implications associated with renal mineralization in chronic kidney disease in cats

**DOI:** 10.1111/jvim.16363

**Published:** 2022-01-19

**Authors:** Pak‐Kan Tang, Rosanne E. Jepson, Yu‐Mei Chang, Rebecca F. Geddes, Mark Hopkinson, Jonathan Elliott

**Affiliations:** ^1^ Department of Comparative Biomedical Sciences Royal Veterinary College, University of London London United Kingdom; ^2^ Department of Clinical Science and Services Royal Veterinary College, University of London London United Kingdom; ^3^ Research Support Office Royal Veterinary College, University of London London United Kingdom

**Keywords:** calcification, CKD‐MBD, hypercalcemia, nephrocalcinosis

## Abstract

**Background:**

Nephrocalcinosis is a pathological feature of chronic kidney disease (CKD). Its pathophysiological implications for cats with CKD are unexplored.

**Objectives:**

Identify nephrocalcinosis risk factors and evaluate its influence on CKD progression and all‐cause mortality.

**Animals:**

Fifty‐one euthyroid client‐owned cats with International Renal Interest Society (IRIS) stages 2‐3 azotemic CKD.

**Methods:**

Retrospective cohort study. Histopathological kidney sections were assessed for nephrocalcinosis (von Kossa stain). Nephrocalcinosis severity was determined by image analysis (ImageJ). Ordinal logistic regressions were performed to identify nephrocalcinosis risk factors. The influence of nephrocalcinosis on CKD progression and mortality risk were assessed using linear mixed model and Cox regression, respectively. Cats were categorized by their owner‐reported time‐averaged phosphate‐restricted diet (PRD) intake, where PRD comprised ≥50%, 10‐50%, or none of food intake.

**Results:**

Nephrocalcinosis was rated as mild‐to‐severe in 78.4% and absent‐to‐minimal in 21.6% of cases. Higher baseline plasma total calcium concentration (tCa; odds ratio [OR] = 3.07 per 1 mg/dL; *P* = .02) and eating a PRD (10%‐50%: OR = 8.35; *P* = .01; ≥50%: OR = 5.47; *P* = .01) were independent nephrocalcinosis risk factors. Cats with absent‐to‐minimal nephrocalcinosis had increasing plasma creatinine (0.250 ± 0.074 mg/dL/month; *P* = .002), urea (5.06 ± 1.82 mg/dL/month; *P* = .01), and phosphate (0.233 ± 0.115 mg/dL/month; *P* = .05) concentrations over a 1‐year period, and had shorter median survival times than cats with mild‐to‐severe nephrocalcinosis.

**Conclusion and Clinical Importance:**

Higher plasma tCa at CKD diagnosis and PRD intake are independently associated with nephrocalcinosis. However, nephrocalcinosis is not associated with rapid CKD progression in cats.

**Abbreviations**
3D: 3‐dimensional
ALP: alkaline phosphatase
ALT: alanine aminotransferase
ANOVA: analysis of variance
Ca:P: calcium‐to‐phosphorus ratio
CaOx: calcium oxalate
CaP: calcium phosphate
CaPP: calcium phosphate product
CI: confidence interval
CKD: chronic kidney disease
CKD‐MBD: chronic kidney disease‐mineral and bone disorder
CT: computed tomography
CTAn: CT‐Analyzer
EDTA: ethylene‐diaminetetraacetic acid
FFPE: formalin‐fixed paraffin‐embedded
FGF23: fibroblast growth factor 23
GFR: glomerular filtration rate
HR: hazard ratio
iCa: ionized calcium
IRIS: International Renal Interest Society
MST: median survival time
OR: odds ratio
PRD: phosphate‐restricted diet
PTH: parathyroid hormone
SBP: systolic blood pressure
tCa: total calcium
TT4: total thyroxine
USG: urine specific gravity
VN:KT: volume of nephrocalcinosis‐to‐kidney tissue ratio

## 1 INTRODUCTION

Disturbances in mineral and bone metabolism are present in cats with chronic kidney disease (CKD), even in the early stages of disease.^1^, ^2^ The kidneys play a fundamental role in calcium and phosphate homeostasis. A gradual decrease in functioning nephrons in CKD results in phosphate retention,^3^ stimulating phosphaturic hormones, fibroblast growth factor 23 (FGF23) and subsequently parathyroid hormone (PTH), in order to maintain physiological plasma phosphate concentrations.^4^, ^5^, ^6^ However, not only does this adaptive response have an influence on calcium metabolism regulation, but also on bone remodeling. When this adaptive response eventually fails to prevent plasma phosphate concentration from increasing, it promotes ectopic calcification. These adaptive responses are collectively referred to as chronic kidney disease‐mineral and bone disorder (CKD‐MBD).^7^

Nephrocalcinosis is characterized by tubulointerstitial calcium phosphate (CaP) or calcium oxalate (CaOx) crystal deposition,^8^ in humans almost exclusively in the renal medulla.^9^ This process begins from Randall's plaques formation in the renal papillae, which act as a nadir for progressive calcification.^9^, ^10^ Observation of this phenomenon on light microscopy is termed microscopic nephrocalcinosis (referred to herein as nephrocalcinosis).^8^, ^11^ Spontaneous ectopic calcification can be explained, in part, by calcium and phosphate salts precipitating from supersaturated fluid when the calcium phosphate product (CaPP) exceeds the solubility product. Previous studies showed that increased serum phosphate concentration and CaPP correlated positively with renal calcium content,^12^, ^13^ and increased serum calcium concentration was an independent risk factor for nephrocalcinosis in human CKD patients.^11^ Nephrocalcinosis is prevalent in humans and cats with CKD.^14^, ^15^ Renal calcium deposition was found on histology in ≥50% of cats with International Renal Interest Society (IRIS) Stage 2 to 4, compared to 21% in non‐azotemic cats.^15^ In vivo in rats, renal calcium content was negatively correlated with creatinine clearance, suggesting a deleterious impact on renal function.^13^, ^16^

Cats with CKD are at increased risk of developing plasma total hypercalcemia, with prevalence increasing with advancing azotemia.^17^ In CKD cats eating a phosphate‐restricted diet (PRD), increasing plasma phosphate and total calcium (tCa) concentrations are associated with CKD progression.^18^ However, it remains to be determined whether calcium and phosphate homeostasis dysregulation plays a role in nephrocalcinosis pathogenesis. Furthermore, the implications of nephrocalcinosis associated with CKD in cats have not been investigated. Our study objectives were to: (a) explore risk factors for nephrocalcinosis in CKD cats and (b) assess associations of nephrocalcinosis with change in CKD‐MBD parameters, CKD progression, and all‐cause mortality.

## 2 METHODS

### 2.1 Case selection

Records of 2 London‐based first‐opinion practices between 1 January 1992 and 31 December 2017 were reviewed and azotemic CKD cats that underwent complete necropsy examinations identified. Azotemic CKD diagnosis was defined as a plasma creatinine concentration ≥2 mg/dL with a urine specific gravity (USG) <1.035, or plasma creatinine concentration ≥2 mg/dL on 2 consecutive occasions 2‐4 weeks apart without evidence of a pre‐renal cause. All cats with a CKD diagnosis were offered a PRD. A variety of PRD were used throughout the study (Feline Low Protein Diet [wet]; Masterfoods, Bruck, Austria; Waltham Veterinary Diet, Feline Low Phosphorus Low Protein [dry and wet]; Effem, Minden, Germany [dry] and Masterfoods, Bruck, Austria [wet]; Feline Veterinary Diet Renal [dry and wet], Royal Canin SAS, Aimargues, France [dry] and Masterfoods, Bruck, Austria [wet]) with a phosphorus content of 0.7‐1.1 g/Mcal and calcium‐to‐phosphorus ratio (Ca:P) of 1.3‐2.1. Cats not accepting a PRD continued on their maintenance diets.

Inclusion required a formalin‐fixed paraffin‐embedded (FFPE) kidney block for histopathological evaluation. Cats were excluded if they had clinically suspected hyperthyroidism and their plasma total thyroxine (TT4) concentration was >40 nmol/L, they were being medically managed for hyperthyroidism, had evidence of other concurrent disease, or were being treated with corticosteroids, furosemide, or bisphosphonates. Cats with IRIS CKD stage 4 at diagnosis and cats with no follow‐up visit after CKD diagnosis also were excluded. Cats receiving amlodipine besylate for systemic hypertension were included.

### 2.2 Clinicopathological data

Blood, urine, and necropsy samples were collected with owner informed consent and Royal Veterinary College Ethics and Welfare Committee approval (URN20131258E). Blood samples were collected by jugular venipuncture into heparinized and ethylene‐diaminetetraacetic acid (EDTA) tubes and urine was obtained by cystocentesis. Samples were stored at 4°C for <6 hours before centrifugation and separation. Heparinized plasma was analyzed biochemically at an external laboratory (IDEXX laboratories, Wetherby, UK). In‐house urinalyses, including USG measurement by refractometry, dipstick chemical analysis, and microscopic urine sediment examination, were performed on the day of collection. Urinary tract infection was confirmed by bacterial culture (Royal Veterinary College Diagnostic Laboratory Services, Hatfield, UK).

Systolic blood pressure (SBP) measurements were made as previously described by Doppler.^19^ Indirect ophthalmoscopy was performed using a retinal camera (ClearView, Optibrand, Fort Collins, Colorado) in cats with an average SBP >160 mm Hg. Systemic hypertension was defined as an average SBP >160 mm Hg in conjunction with ocular pathology consistent with hypertensive damage, or SBP >170 mm Hg on 2 consecutive occasions.

Clinical records were reviewed to extract the following: age, sex, breed, body weight, SBP, tCa, ionized calcium (iCa), plasma creatinine, urea, phosphate, potassium, sodium, chloride, total protein, albumin and TT4 concentrations, plasma alanine aminotransferase (ALT) and alkaline phosphatase (ALP) activities, PCV, USG, and urine culture results. The proportion of PRD recorded as being fed at every visit (until death) from each cat with PRD prescribed after CKD diagnosis was reviewed from our clinical records. Owners were asked at each visit to estimate the proportion of PRD by volume of the total quantity of food fed. A time‐averaged proportion of PRD fed by volume of the total ration was calculated from this estimate for each cat. Where the proportion fed was missing from the record at a particular visit, the proportion stated at the previous visit was imputed, except if it was the visit at which euthanasia was recommended and the cat had deteriorated clinically from the previous visit. Cats were categorized according to the time‐averaged ingestion of PRD, where PRD comprised ≥50%, >10%, <50%, or none of their food intake for their CKD duration.

### 2.3 Histopathological data

**FIGURE 1:**
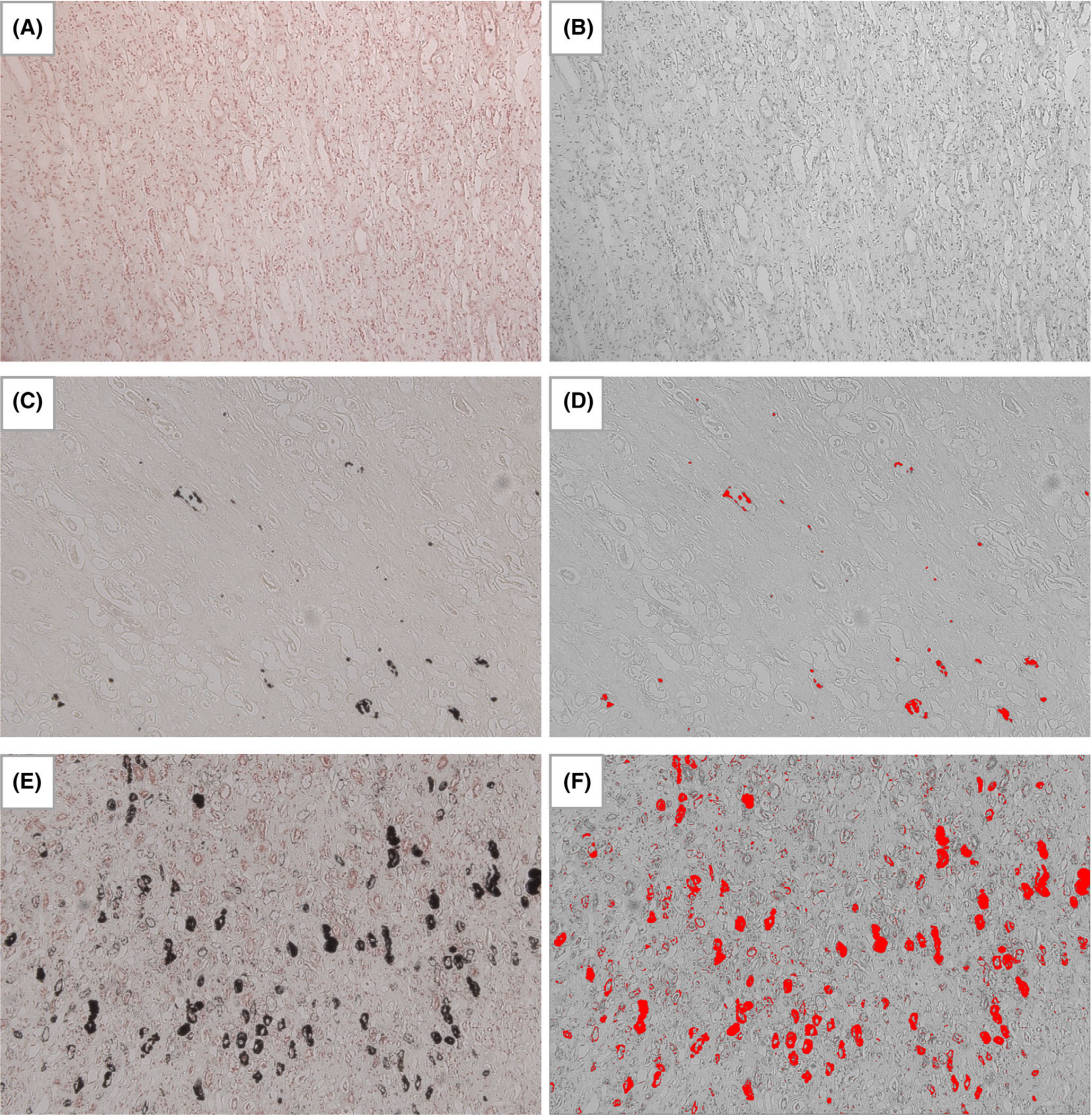
Kidney, von Kossa stain, ×10 magnification. An example of each nephrocalcinosis grade (0‐2) is shown as follows: (A) and (B) cat no. 7 with grade 0 nephrocalcinosis (0.004%); (C) and (D) cat no. 34 with grade 1 nephrocalcinosis (0.327%); (E) and (F) cat no. 39 with grade 2 nephrocalcinosis (5.51%). Von Kossa positive staining is outlined in black coloring in the original images (A, C, and E), or bright red in color after processing using ImageJ (B, D, and F; 8‐bit color; threshold 0‐92) for the quantification of the proportional area of nephrocalcinosis in each case (n = 51)

Necropsy examinations were offered to all clients when their cats were euthanized and informed consent was obtained from those who agreed. At necropsy, each kidney was bissected longitudinally and transversely and fixed (10% neutral buffered formalin). Formalin‐fixed renal tissues (including cortex and medulla) were paraffin embedded for future histopathological evaluation. A 4‐μm section of FFPE tissue was stained with von Kossa to identify hydroxyapatite deposits.^20^ Stained sagittal kidney sections were imaged, blinded to case data, using a digital microscope (Leica DM4000, Wetzlar, Germany) for microscopic nephrocalcinosis assessment (Figure 1). ImageJ (Version 2.1.0, National Institutes of Health, Bethesda, Maryland) was used to quantify nephrocalcinosis from representative images captured at the medullary region. Nephrocalcinosis was graded according to the average proportion of positively stained tissue from 5 medullary images captured at ×10 magnification. They were graded as follows: <0.06% = grade 0, 0.06% to 1% = grade 1, and >1% = grade 2 (Figure 1). This grading system, pre‐determined before statistical analyses, provided an objective quantification of nephrocalcinosis severity (0.06% and 1% are equivalent to 1 × 10^−5^ cm^2^ and 1.4 × 10^−4^ cm^2^, respectively). An adjacent FFPE section from each case was stained with Alizarin red (at pH 4.2), allowing CaP and CaOx crystal differentiation.^20^

### 2.4 Micro‐computed tomography (micro‐CT) for macroscopic nephrocalcinosis

The FFPE kidney blocks were scanned for macroscopic nephrocalcinosis using a micro‐computed tomography (CT) scanner (Skyscan 1172, Bruker, Kontich, Belgium) using a small (4000 × 2672 pixels) camera without a filter. Images were obtained using the following scanning parameters: isotropic voxel size 5 μm per pixel, source voltage 50 kV, source current 200 μA, exposure time 670 ms, and imaging rotation scan 180° with a 0.4° rotation step. Projection images were reconstructed into tomograms using NRecon 1.7.5.9 (Bruker, Kontich, Belgium) and repositioned using Dataviewer 1.5.6.6 (Bruker, Kontich, Belgium). Tomograms were analyzed using Bruker analysis software CT‐Analyzer (CTAn) 1.20.3 (Bruker, Kontich, Belgium) and volume‐rendered 3‐dimensional (3D) visualizations were created using CTVox 3.3 (Bruker Kontich, Belgium). Briefly, for each block, tissue volume was calculated by multiplying the depth by the average area from 5 tomograms spaced at equal distances throughout the sample; nephrocalcinosis volume was calculated using the 3D analysis tool in CTAn. The nephrocalcinosis volume‐to‐kidney tissue ratio (VN:KT) was calculated using the formula:

$$\mathrm{VN}:\mathrm{KT}\;\left(\%\right)=\frac{\text{Volume of nephrocalcinosis}}{\text{Volume of kidney tissue}}\times 100\%$$

### 2.5 Statistical analysis

Statistical analyses were performed using R software (Version 4.1.1 GUI 1.77 High Sierra build, R Foundation for Statistical Computing, Vienna, Austria). Type I error rate was set at .05. Continuous variables were assessed for normality by visual inspection of histograms and using the Shapiro‐Wilk test. Levene's test was used to test if the groups had equal variances. Most data were not normally distributed and therefore numerical data are presented as median (25th, 75th percentile) for consistency. Categorical data are presented as percentages.

#### 2.5.1 Nephrocalcinosis risk factors

Baseline variables were compared between groups by either 1‐way analysis of variance (ANOVA) followed by Tukey *post‐hoc* test or Kruskal‐Wallis and Dunn's *post‐hoc* test for continuous variables with normal or skewed distributions, respectively. Proportions of categorical outcomes were compared using Fisher's exact test.

Baseline microscopic nephrocalcinosis risk factors were assessed using ordinal logistic regression. Age, bodyweight, tCa, creatinine, urea, phosphate, potassium, sodium, chloride, total protein, albumin, ALT, ALP, PCV, USG, and CKD survival time were entered as continuous variables, whereas sex and proportion of PRD ingested (“Not eating PRD” vs “Eating 10%–50% PRD” vs “Eating ≥50% PRD”) were entered as categorical variables for univariable analyses. Variables associated with nephrocalcinosis at *P* < .10, and with data available for at least half of the cats (n > 25), were entered into a multivariable model. Manual backward elimination was applied to obtain the final model with *P* < .05. Ordinal version of the Hosmer‐Lemeshow test was used to evaluate the final model's goodness‐of‐fit, and presence of co‐linearity among the significant independent risk factors (*P* < .05) was assessed by variance inflation factor. Residual for outliers and influential observations were checked by Quantile‐Quantile plot visual inspection. Linear relationship between continuous predictor and the logit was assessed by categorizing the variables into equal intervals of “low,” “medium,” and “high” in the logistic regression analysis and evaluating the increase or decrease trend of the coefficient. Results are reported as odds ratio (OR; 95% confidence interval [CI]).

#### 2.5.2 Changes in CKD‐MBD parameters over time in relation to nephrocalcinosis

Linear mixed effects models were used to assess changes in continuous clinicopathological variables over time. Longitudinal data from all available visits during the first 365 days after CKD diagnosis were included for the following: bodyweight, tCa, creatinine, urea, phosphate, potassium, sodium, chloride, total protein, albumin, ALT, ALP, and PCV. Group (“grade 0” vs “grade 1” vs “grade 2”), time (in months [30.4 days]) and the interaction between group and time were treated as fixed effects. Each cat's case number and time nested within individual cats were included as 2 uncorrelated random effects. Residuals were assumed to be independent in the model, and normality was checked. No attempt was made to impute missing data. Results are reported as coefficient (β) ± SE.

#### 2.5.3 Association of nephrocalcinosis and other factors with survival

Date of azotemic CKD diagnosis was defined as baseline, whereas death of all‐cause was the event of interest. Survival times were depicted with a Kaplan‐Meier curve and were compared among groups using log‐rank test and Kruskal‐Wallis with Dunn's *post‐hoc* tests because all cats reached study endpoint. Baseline variables associated with survival were explored using Cox proportional hazard analysis. Martingale residuals were used to assess the assumption of linearity of the continuous variables in the Cox model. Residuals for outliers and influential observations were checked by Quantile‐Quantile plot visual inspection. Continuous variables were transformed into categorical variables based on tertiles (age, sodium) if the assumption of proportional hazards, as evaluated by Kaplan‐Meier curve inspection, and assessment of the independence between each variable and time were not met. Variables associated with survival with *P* < .10 in univariable analyses were entered into a multivariable Cox model. The final Cox regression model was derived by manual backward elimination with *P* < .05. Results are reported as hazard ratio (HR; 95% CI).

#### 2.5.4 von Kossa with Alizarin red staining correlation and macroscopic nephrocalcinosis

For each case, proportional nephrocalcinosis areas in renal medulla at ×2.5 magnification, as stained by von Kossa and Alizarin red separately, were calculated. The relationship between these 2 staining techniques for microscopic nephrocalcinosis was evaluated using Spearman's correlation. In a subset of 49 cases, macroscopic nephrocalcinosis was assessed using micro‐CT. Spearman's correlation was used to evaluate the relationship between proportional nephrocalcinosis volume (micro‐CT) and proportional nephrocalcinosis area (von Kossa, overview ×0.14‐×0.36 magnification).

## 3 RESULTS

### 3.1 Risk factors associated with nephrocalcinosis

A total of 122 CKD cats with necropsy examinations were identified between 1 January 1992 and 31 December 2017, of which 32 were excluded because of suspected or documented hyperthyroidism (n = 28), diabetes mellitus (n = 1), or corticosteroid (n = 2) or furosemide (n = 1) administration. Of the remaining 90 cats, 39 were excluded because of advanced CKD (IRIS stage 4; [n = 19]) diagnosis, renal neoplasia (n = 3) or renal dysplasia (n = 1), insufficient follow‐up (n = 9), missing FFPE tissues (n = 3), or lack of medullary region (n = 4) for nephrocalcinosis assessment. In total, 51 CKD cats (IRIS stage 2, n = 34; IRIS stage 3, n = 17) were enrolled, with 11 having absent to minimal nephrocalcinosis (grade 0), 14 having mild to moderate nephrocalcinosis (grade 1), and 26 with severe nephrocalcinosis (grade 2). Seventy‐one percent (n = 36) of CKD cats were managed by PRD feeding, of which 67% (n = 24) had eaten ≥50% of PRD (76.5 [64.9, 91]%), whereas the remaining 33% (n = 12) had eaten 10% to 50% of PRD (31 [15.7, 35.3]%). Twenty‐nine percent (n = 15) did not accept PRD and continued consuming maintenance diets.

Domestic shorthair was the most common breed (n = 40), followed by domestic longhair (n = 7), Burmese (n = 2), Siamese (n = 1), and Persian cross (n = 1). Baseline clinicopathological variables are summarized in Table 1. Baseline plasma tCa concentration was significantly higher in cats with grade 2 nephrocalcinosis than in cats with grade 0 nephrocalcinosis (*P* = .04). No other significant differences in clinicopathological variables were identified. Table 2 presents baseline univariable risk factors associated with nephrocalcinosis. In the final multivariable model, baseline tCa concentration (OR = 3.07 [95% CI: 1.26‐8.40] per 1 mg/dL increase; *P* = .02) and ingestion of 10% to 50% (OR = 8.35 [95% CI: 1.75‐47.64]; *P* = .01) and ≥50% PRD (OR = 5.47 [95% CI: 1.50‐21.78]; *P* = .01) remained nephrocalcinosis independent risk factors (Table 2).

**TABLE 1:** Descriptive statistics for the baseline data collected from 51 cats enrolled in this retrospective cohort study, grouped according to the quantification of calcium deposition in the kidneys using von Kossa stain and an imaging processing program (ImageJ)

Variables (reference interval)	Grade 0 (n = 11)		Grade 1 (n = 14)		Grade 2 (n = 26)		*P*‐value
	Median (25th, 75th percentile)	n	Median (25th, 75th percentile)	n	Median (25th, 75th percentile)	n	
Age (years)	14.0 (9.5, 16.0)	11	14.2 (13.0, 15.8)	14	16.1 (13.8, 17.0)	26	.18
Phosphate‐restricted diet (Eating ≥50%), n (%)	3 (27)	11	6 (43)	14	14 (54)	26	.08
Sex (female neutered), n (%)	2 (18)	11	9 (64)	14	11 (42)	26	.07
Weight (kg)	4.3 (3.5, 5.5)	10	3.7 (3.2, 4.1)	13	3.9 (3.3, 4.4)	24	.18
Albumin (2.5‐4.5 g/dL)	2.8 (2.7, 3.2)	11	3.1 (3.0, 3.4)	14	3.1 (3.0, 3.3)	26	.15
ALP (≤ 60 U/L)	50 (35, 89)	11	38 (25, 58)	14	28 (20, 52)	26	.13
ALT (5‐60 U/L)	73 (47, 122)	11	53 (44, 75)	14	61 (47, 73)	26	.54
CaPP (<70 mg^2^/dL^2^)	49.0 (41.9, 82.8)	11	41.2 (30.7, 60.4)	14	42.5 (38.5, 55.3)	26	.28
Chloride (100‐124 mEq/L)	116 (113, 119)	11	116 (113, 119)	14	118 (115121)	26	.22
Creatinine (0.23‐2 mg/dL)	2.79 (2.46, 3.31)	11	2.58 (2.14, 2.90)	14	2.48 (2.30, 3.04)	26	.25
Hypertension (controlled), n (%)	4 (67)	6	6 (60)	10	11 (48)	23	.74
Ionized calcium (4.76‐5.48 mg/dL)	4.94 (4.65, 5.17)	4	5.18 (5.00, 5.27)	6	5.44 (5.30, 5.44)	3	.22
PCV (30‐45%)	30 (26, 35)	11	36 (33, 40)	12	34 (30, 35)	23	.05
Phosphate (2.79‐6.81 mg/dL)	5.33 (4.27, 8.22)	11	3.98 (3.02, 6.08)	14	4.46 (3.78, 5.31)	26	.12
Potassium (3.5‐5.5 mEq/L)	4.00 (3.74, 4.27)	11	4.21 (3.76, 4.48)	14	4.20 (3.89, 4.51)	26	.64
SBP (<160 mmHg)	179 (154, 195)	6	161 (133, 189)	10	146 (132, 171)	23	.16
Sodium (145‐157 mEq/L)	150 (148, 152)	11	149 (150, 152)	14	152 (151, 156)	26	.05
Total calcium (8.2‐11.8 mg/dL)	9.48 (9.32, 9.94)	11	10.14 (9.95, 10.68)	14	10.28 (9.84, 10.67)	26	**.04**
Total protein (6.0‐8.0 g/dL)	7.7 (7.5, 7.8)	11	7.7 (7.2, 8.1)	14	7.8 (7.4, 8.3)	26	.70
Urea (7.0‐27.7 mg/dL)	46.8 (41.0, 69.8)	11	47.1 (38.3, 54.6)	14	49.4 (42.8, 59.7)	26	.71
USG (≥1.035)	1.022 (1.015, 1.026)	9	1.018 (1.015, 1.023)	13	1.016 (1.014, 1.023)	22	.65

*Note*: Significant difference between groups (*P* < .05) is highlighted in bold.

Abbreviations: ALP, alkaline phosphatase; ALT, alanine aminotransferase; CaPP, calcium phosphate product; n, number of cats; SBP, systolic blood pressure; USG, urine specific gravity.

**TABLE 2:** Univariable and multivariable backward ordinal logistic regression to identify risk factors at baseline associated with nephrocalcinosis in CKD cats

Variables	Univariable analysis			Multivariable analysis		
	OR (95% CI)	n	*P*‐value	OR (95% CI)	n	*P*‐value
Age (years)	1.11 (0.97‐1.27)	51	.09			
Chloride (mEq/L)	1.11 (0.99‐1.25)	51	.09			
Phosphate‐restricted diet		51			51	
Eating 0%	NA		NA	NA		NA
Eating 10% to 50%	5.11 (1.19‐24.51)		.03	8.35 (1.75–47.64)		.01
Eating ≥50%	5.54 (1.55‐21.44)		.01	5.47 (1.50‐21.78)		.01
Phosphate (mg/dL)	0.77 (0.58‐1.01)	51	.06			
Sodium (mEq/L)	1.23 (1.04‐1.48)	51	.02			
Total calcium (mg/dL)	2.51 (1.11‐6.23)	51	.04	3.07 (1.26–8.40)	51	.02

Abbreviations: CI, confidence interval; OR, odds ratio.

### 3.2 Changes in clinical variables over time in relation to nephrocalcinosis

**FIGURE 2:**
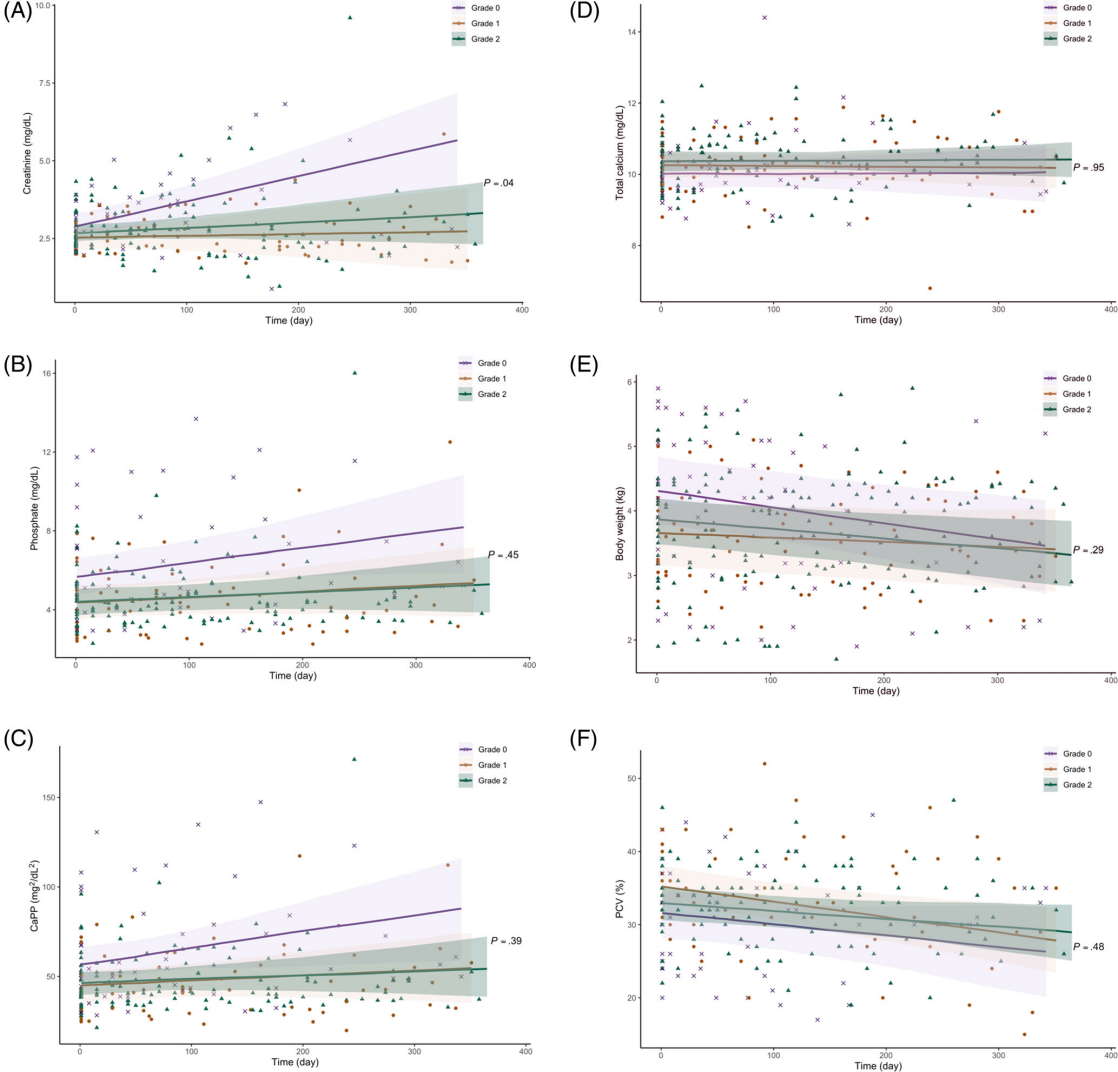
Scatter plots illustrating the linear change of (A) creatinine, (B) phosphate, (C) CaPP, (D) total calcium, (E) bodyweight, and (F) PCV in CKD cats grouped according to the severity of nephrocalcinosis (“grade 0” vs “grade 1” vs “grade 2”) during the first 365 days after transition to a phosphate‐restricted diet. The solid lines represent the regression lines and the shaded areas represent 95% confidence interval (95% Cl) for the fitted linear regression. The *P*‐values shown are the interactions among groups and the rate of change for these analyses within groups are presented in Table S1

Changes in clinicopathological variables over a 1‐year period (365 days) after CKD diagnosis were assessed using a linear mixed model (Tables 3 and S1). The unit of time was expressed as month (30.4 days). Both plasma creatinine and urea concentrations increased significantly over time in cats with grade 0 nephrocalcinosis (creatinine, *β*, 0.250 ± 0.074 mg/dL; *P* = .002; urea, *β*, 5.06 ± 1.82 mg/dL; *P* = .01). These laboratory results did not change significantly over time in grade 1 (creatinine, *β*, 0.017 ± 0.056 mg/dL; *P* = .77; urea, *β*, 1.00 ± 1.34 mg/dL; *P* = .46) and grade 2 cats (creatinine, *β*, 0.050 ± 0.042 mg/dL; *P* = .24; urea, *β*, 2.04 ± 1.07 mg/dL; *P* = .06; Figure 2A). In addition, cats with grade 0 nephrocalcinosis had significantly increasing plasma phosphate concentration and CaPP over time (phosphate, *β*, 0.233 ± 0.115 mg/dL; *P* = .05; CaPP, *β*, 2.70 ± 1.29 mg^2^/dL^2^; *P* = .04), whereas these variables did not change significantly for those with grade 1 (phosphate, *β*, 0.089 ± 0.079 mg/dL; *P* = .27; CaPP, *β*, 0.87 ± 0.89 mg^2^/dL^2^; *P* = .34) and grade 2 nephrocalcinosis (phosphate, *β*, 0.068 ± 0.064 mg/dL; *P* = .29; CaPP, *β*, 0.69 ± 0.71 mg^2^/dL^2^; *P* = .34; Figure 2B, C). Plasma tCa concentration did not change significantly over time among the 3 groups (Figure 2D). The bodyweight of cats with grade 0 (*β*, −0.07 ± 0.03 kg; *P* = .01) and grade 2 nephrocalcinosis (*β*, −0.04 ± 0.02 kg; *P* = .01) decreased significantly over time but did not change significantly over time in grade 1 cats (*β*, −0.02 ± 0.02 kg; *P* = .24; Figure 2E). The PCV in cats with grade 1 (*β*, −0.62 ± 0.19%; *P* = .002) and grade 2 nephrocalcinosis (*β*, −0.33 ± 0.15%; *P* = .04) decreased significantly, whereas it did not change overtime in cats with grade 0 nephrocalcinosis (*β*, −0.49 ± 0.27%; *P* = .08; Figure 2F).

**TABLE 3:** Linear mixed model analyses examining the rate of change in CKD‐MBD variables over the first 365 days after a diagnosis of azotemic CKD in cats (n = 51)

Variables	Group	Time	Group × Time
Body weight (kg)	.22	**<.01**	.30
Albumin (g/dL)	.29	.59	.29
ALP (U/L)	.73	.40	.71
ALT (U/L)	.57	.85	.34
CaPP (mg^2^/dL^2^)	.19	**.02**	.39
Chloride (mEq/L)	.38	.78	.44
Creatinine (mg/dL)	.35	**<.01**	**.04**
PCV (%)	.25	**<.01**	.48
Phosphate (mg/dL)	.10	**.02**	.45
Potassium (mEq/L)	.67	.85	.50
Sodium (mEq/L)	.13	.37	.16
Total calcium (mg/dL)	.34	.98	.95
Total protein (g/dL)	.98	.08	.73
Urea (mg/dL)	.55	**<.01**	.21

*Note*: Summary of *P*‐values for all variables included in the model. Group represents cats in “grade 0,” “grade 1,” or “grade 2” based on the severity of nephrocalcinosis. Outcome variables showing significant change over time and among groups (*P* < .05) are highlighted in bold. The unit used for time was month (30.4 days). A significant difference in the group column indicates a significant difference among the 3 groups at baseline for a given parameter (the start of the regression line at time 0). A significant difference in Group × Time indicates that the outcome variable differs significantly among groups (“grade 0” vs “grade 1” vs “grade 2”) over time. If Group × Time was not significant, a significant difference in Time indicates the overall rate of change of the outcome variable differs significantly from baseline over time. See Table S1 for a summary of intercepts and the slopes of time within each group.

Abbreviations: ALP, alkaline phosphatase; ALT, alanine aminotransferase; CaPP, calcium phosphate product.

### 3.3 Nephrocalcinosis and other factor association with survival

**FIGURE 3:**
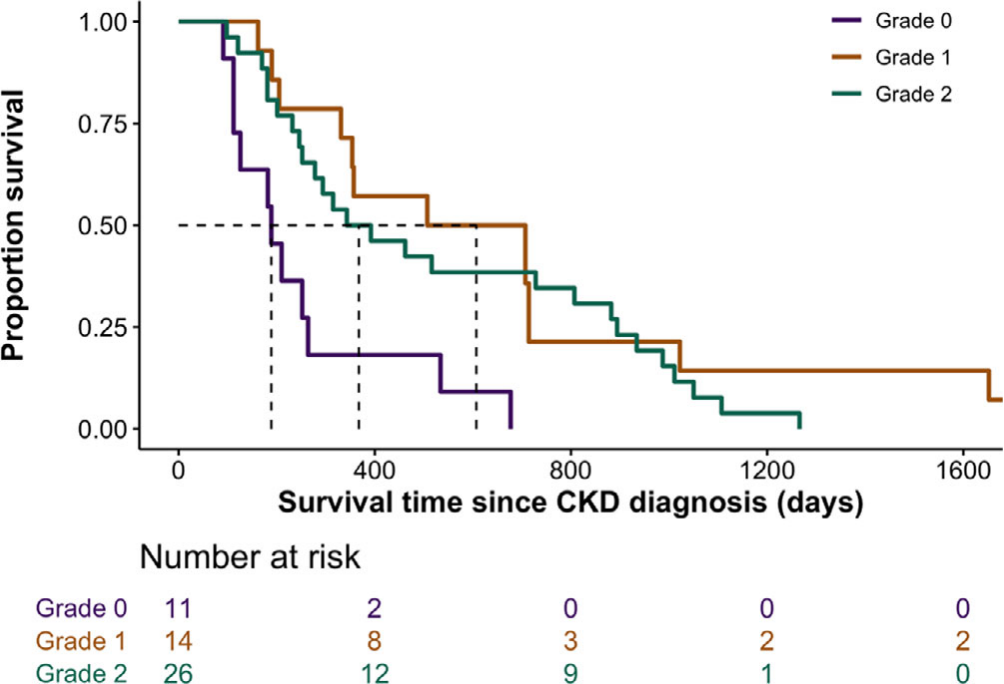
Kaplan–Meier curve illustrating survival in cats with CKD (n = 51) grouped by the severity of nephrocalcinosis (“grade 0” vs “grade 1” vs “grade 2”). No censoring was required as all cats reached study endpoint. Cats with grade 0 nephrocalcinosis had significantly shorter median survival times (189 [119, 258] days) than cats with grade 1 (607 [337, 714] days; *P* = .01) and grade 2 nephrocalcinosis (368 [236, 891] days; *P* = .02)

**FIGURE 4:**
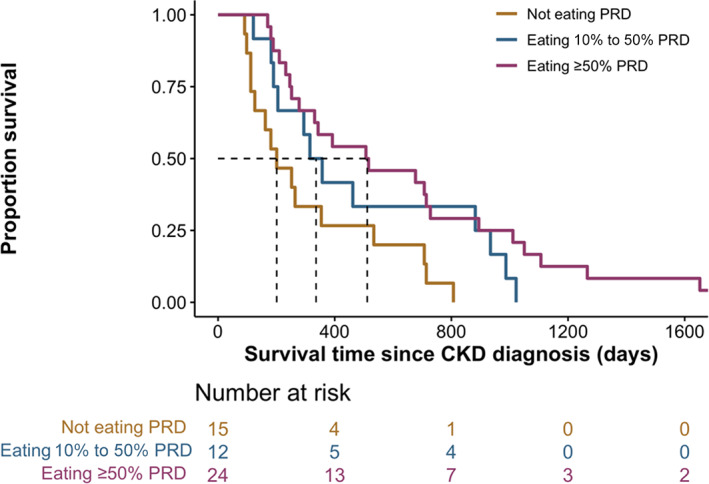
Kaplan‐Meier curve illustrating survival in cats with CKD (n = 51) grouped by the proportion of PRD ingested (“Not eating PRD” vs “Eating 10% to 50% PRD” vs Eating ≥50% PRD). No censoring was required as all cats reached study endpoint. Cats that had eaten ≥50% PRD had significantly longer median survival times (512 [251, 923] days) than cats that refused to eat a PRD (201 [119, 444] days; *P* = .01)

Risk of death within a year of azotemic CKD diagnosis was 54.9% (n = 28) for the entire population, with 81.8% (n = 9), 42.9% (n = 6), and 50% (n = 13) for cats with grades 0, 1, and 2 nephrocalcinosis, respectively. A significantly shorter median survival time (MST) was found in cats with grade 0 (189 [119, 258] days) compared to cats with grade 1 (607 [337, 714] days; *P* = .01) and grade 2 nephrocalcinosis (368 [236, 891] days; *P* = .02; Figure 3). Baseline variables associated with survival time identified at the 10% level (*P* < .1) in univariable Cox regression analyses are shown in Table 4. Survival curves for the different diet groups are presented in Figure 4. In the final multivariable Cox regression model after adjustment for confounders, risk of death was positively associated with baseline plasma phosphate concentration (HR = 1.23 [95% CI, 1.03‐1.48]; *P* = .03) and negatively associated with ingestion of ≥50% PRD (HR = 0.36 [95% CI, 0.17‐0.75]; *P* = .01) and baseline PCV (HR = 0.91 [95% CI, 0.84‐0.98]; *P* = .01; Table 4).

**TABLE 4:** Univariable and multivariable backward Cox regression analysis of factors at baseline associated with risk of death (all‐cause mortality) in CKD cats

Variables	Univariable analysis			Multivariable analysis		
	HR (95% CI)	n	*P*‐value	HR (95% CI)	n	*P*‐value
ALT (U/L)	1.00 (1.00‐1.00)	51	.04			
Chloride (mEq/L)	0.93 (0.88‐1.00)	51	.04			
Phosphate‐restricted diet		51			46	
Eating 0%	NA		NA	NA		NA
Eating 10% to 50%	0.51 (0.24‐1.12)		.09	NA		NA
Eating ≥50%	0.34 (0.17‐0.69)		<.01	0.36 (0.17–0.75)		.01
PCV (%)	0.90 (0.85‐0.96)	46	<.01	0.91 (0.84–0.98)	46	.01
Phosphate (mg/dL)	1.35 (1.15‐1.58)	51	<.01	1.23 (1.03–1.48)	46	.03
Total protein (g/dL)	1.55 (0.98‐2.46)	51	.06			
Urea (mg/dL)	1.02 (1.00‐1.04)	51	.07			
CaPP (mg^2^/dL^2^)	1.03 (1.01‐1.05)	51	<.01			

Abbreviations: ALT, alanine aminotransferase; CaPP, calcium phosphate product; CI, confidence interval; HR, hazard ratio.

### 3.4 Correlation within microscopic nephrocalcinosis and comparison with macroscopic nephrocalcinosis

**FIGURE 5:**
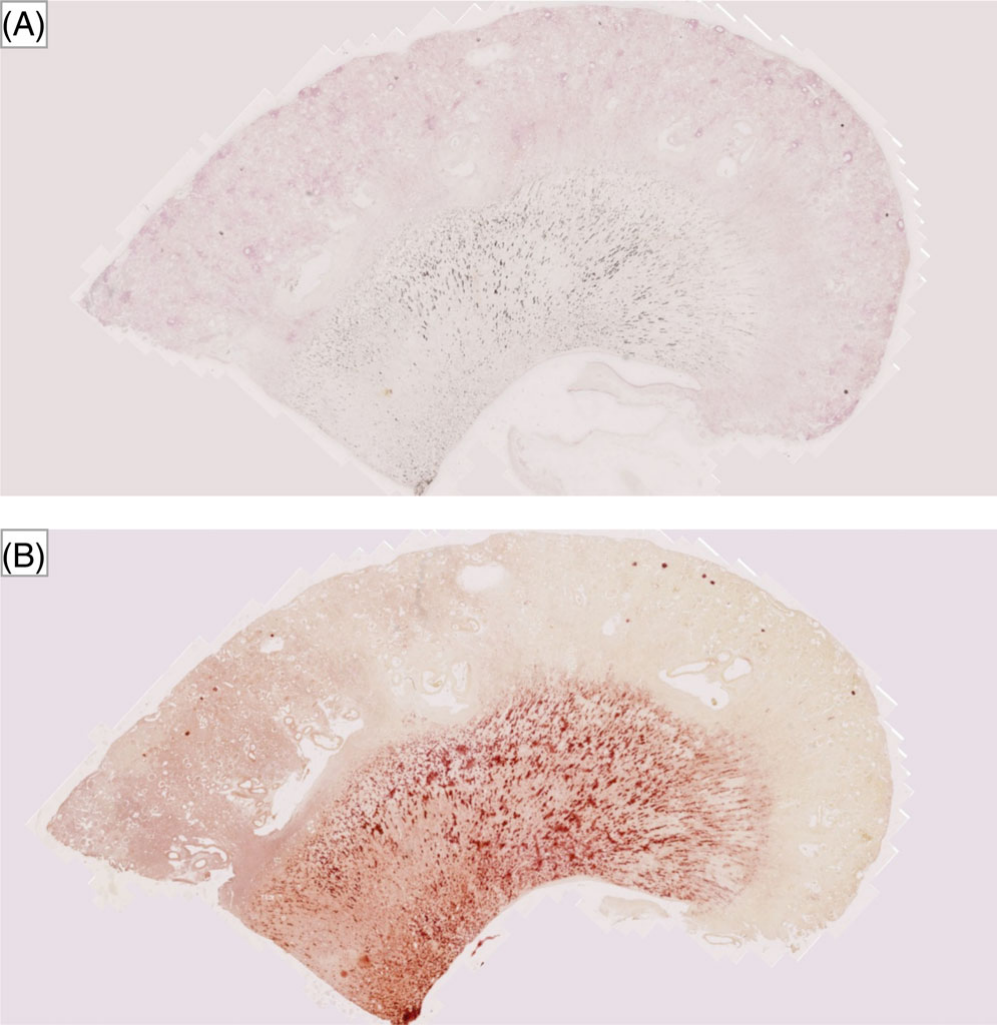
Overview images of a kidney section with grade 2 nephrocalcinosis (cat no. 33) stained with (A) von Kossa and (B) Alizarin Red; positive staining for calcium deposition are indicated by black and red coloring, respectively

**FIGURE 6:**
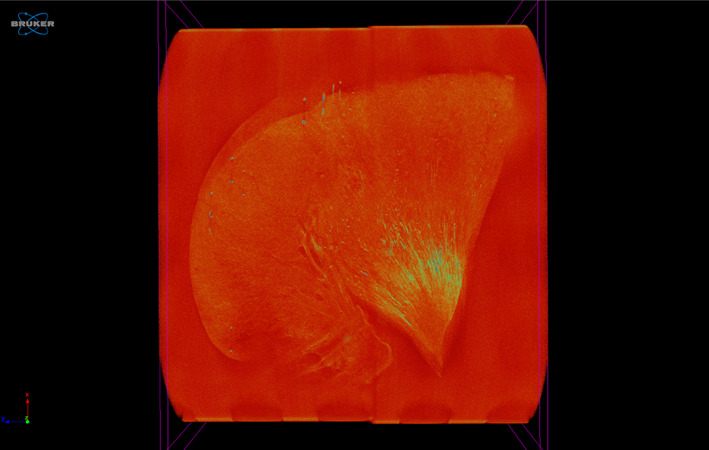
Micro‐computed tomography (micro‐CT) image of a kidney section with grade 2 nephrocalcinosis (cat no. 17), projection images were reconstructed into tomograms and volume‐rendered 3‐dimensional visualizations were created using Bruker micro‐CT SkyScan software (NRecon and CTVox, respectively). Renal calcification is outlined in yellow‐green coloring

A strong correlation was identified between the proportional nephrocalcinosis area detected by von Kossa and Alizarin red staining at ×2.5 magnification (n = 51, *r*_*s*_ = 0.91; *P* < .001; Figure 5). A moderate correlation also was identified between microscopic (von Kossa) and macroscopic nephrocalcinosis, as determined by VN:KT from micro‐CT (n = 49, *r*_*s*_ = 0.6; *P* < .001; Figure 6).

## 4 DISCUSSION

Our results indicated that higher baseline plasma tCa concentration and PRD ingestion were independent nephrocalcinosis risk factors. Cats with grade 0 nephrocalcinosis were associated with increasing plasma creatinine, urea, and phosphate concentrations over the first 365 days after azotemic CKD diagnosis. This cohort of cats also had significantly shorter survival time compared to those with more severe nephrocalcinosis (grades 1 and 2). A significant independent positive association between dietary phosphate restriction and survival was observed.

Renal mineralization is a complicated and multifaceted process and its pathogenesis in association with CKD remains unclear. However, increasing evidence suggests that mineral metabolism disturbances, particularly calcium and phosphate, are likely to contribute to nephrocalcinosis.^11^, ^12^ In our study, higher plasma tCa at CKD diagnosis, even with tCa within the reference interval, was an independent nephrocalcinosis risk factor; only 1 (2%) cat had total hypercalcemia at baseline. This finding suggests that mild perturbations in calcium homeostasis may promote renal mineralization in CKD cats. Consistent with our findings, a CKD study in humans showed that serum tCa concentration was an independent nephrocalcinosis risk factor.^11^ Furthermore, an association between total hypercalcemia and nephrocalcinosis was found in human patients after kidney transplantation,^21^ and patients with macroscopic nephrocalcinosis by CT imaging had higher tCa and iCa concentrations than those without.^22^ However, a histopathological study in humans found no association of serum tCa concentration with renal calcium content and renal tubular calcium deposition.^12^ The discrepancies in calcium involvement in nephrocalcinosis between studies are attributable to the study population heterogenicity, sample size, and renal calcification detection methods.

Ingestion of a PRD was identified as an independent nephrocalcinosis risk factor in CKD cats in our study. Dietary phosphate restriction significantly influences mineral and hormonal regulation in CKD‐MBD including decreases in FGF23 and PTH.^23^, ^24^ A recent study showed that certain CKD cats developed an increasing trend in tCa and iCa concentrations, together with higher urinary calcium excretion, after a PRD initiation.^18^ Lower dietary phosphate content and higher dietary Ca:P ratio, when compared with commercially available foods formulated for healthy adult cats,^25^ potentially could enhance intestinal calcium absorption and increase plasma calcium concentration.^26^ In human patients, hypercalciuria is a common nephrocalcinosis risk factor.^27^ It is postulated, therefore, that nephrocalcinosis may be driven by the increase in plasma tCa concentration and enhanced urinary calcium excretion after transition to a PRD in CKD cats. However, direct evidence supporting this hypothesis could not be obtained in our study and prospective studies measuring urinary electrolytes are required.

Our results showed that nephrocalcinosis is not positively associated with CKD progression and all‐cause mortality. This finding is intriguing, because it is somewhat contrary to a previous study in humans that suggested a detrimental role of nephrocalcinosis on renal function by identifying a positive correlation between renal calcium content and serum creatinine concentration.^12^ Nephrocalcinosis identified by imaging (CT, ultrasonography or radiography) also has been associated with increased risk of end stage kidney disease in human patients.^28^ A study in CKD cats showed renal mineralization association with more severe interstitial inflammation and fibrosis, suggesting its role in accelerating CKD progression.^15^ However, recent cohort studies involving human patients with kidney CT imaging found a lack of association between renal function and renal calcification.^22^, ^29^ This observation is consistent with our results and supports our hypothesis that nephrocalcinosis, to a certain extent, may not exert a direct deleterious effect on kidney function and contribute to CKD progression. Nonetheless, future prospective studies are required to better understand nephrocalcinosis implications for CKD deterioration in cats.

Although PRD ingestion was an independent nephrocalcinosis risk factor, our survival analysis showed that dietary phosphate restriction (when ≥50% PRD was ingested) was associated with longer MST and increased the odds of survival by up to 3‐fold for CKD cats when compared to cats that consumed their maintenance diets (Table 4 and Figure 4). These findings support previous studies that found a survival benefit resulting from dietary phosphate restriction in CKD cats.^30^, ^31^, ^32^ Moreover, results from our sub‐analysis (Tables S2 and S3) showed that CKD cats that continued to be fed maintenance diets had greater increases in plasma creatinine, urea, and phosphate concentrations and decrease in PCV and bodyweight over the first year after CKD diagnosis, further reinforcing current evidence on the salutary effects of dietary phosphate restriction for alleviating disease progression.^23^, ^24^, ^32^, ^33^ Hyperphosphatemia is associated with a decrease in renal function,^34^ and phosphate binders have been shown to protect against the progression of CKD.^35^, ^36^

Plasma phosphate concentration and PCV were independent predictors of all‐cause mortality. Hyperphosphatemia and anemia are associated with more progressive CKD and poorer prognosis in cats and humans.^37^, ^38^, ^39^, ^40^, ^41^, ^42^ Phosphate retention occurs as glomerular filtration rate (GFR) decreases but, in the early stages of CKD, plasma phosphate concentrations usually are maintained within physiological limits as a result of adaptive hormonal regulatory mechanisms, including increased FGF23 and PTH production and decreased calcitriol production.^6^, ^43^ Therefore, higher plasma phosphate concentration at CKD diagnosis could be suggestive of more severely deranged phosphate homeostasis and associated with higher risk of death. Consistent with our results, a previous study found that higher serum phosphate concentration was independently associated with survival in 773 CKD cats.^42^ The pathogenesis of anemia in CKD is multifactorial, but decreased erythropoietin production is a main contributing factor.^42^, ^44^ In support of our findings, other studies also found an association between lower PCV and increased mortality in CKD cats,^38^, ^39^, ^45^ but not all.^42^ Interestingly, neither plasma creatinine concentration nor IRIS staging was a predictor of death in the present study, which is inconsistent with previous studies.^38^, ^39^, ^42^, ^45^, ^46^ Plasma creatinine concentration reflects GFR and is a surrogate biomarker commonly used to assess kidney function.^47^ The discrepancy could be explained by the exclusion of IRIS stage 4 CKD cats in our study, and the comparatively small sample size, with only 17 IRIS stage 3 CKD cats. Additionally, 29% (n = 15) of cats with azotemic CKD in our study did not receive dietary management for CKD, which this may have affected outcome and precluded an accurate assessment of the predictive value of plasma creatinine concentration, and potentially other variables at the time of CKD diagnosis on survival in our study.

Detection of calcium deposition was performed with both the von Kossa method using silver nitrate and Alizarin red staining. The von Kossa method principle is based on the binding of silver ions with anions, such as phosphate, oxalate, and carbonate, from calcified tissues and the reduction of silver salts, leading to the observation of black metallic silver staining.^20^, ^48^, ^49^ Alizarin red staining is another technique used for demonstration of calcium crystals.^50^ It binds directly to calcium ions and can be used to differentiate CaOx from CaP because CaOx can only be stained with Alizarin Red at a pH of 7 but not at pH 4.2, with the latter used in our study.^51^, ^52^ We observed a strong correlation between these 2 nephrocalcinosis staining methods, suggesting that the mineral deposits were primarily composed of CaP. This observation is interesting because the majority of upper urinary tract uroliths in cats contain CaOx.^53^ It may be that precipitation of CaP is a prerequisite for nephrocalcinosis, nephrolithiasis, or both in cats, a process that resembles the formation of Randall's plaques in humans.^8^ Micro‐CT scanning was performed on the FFPE kidney samples to determine whether a single von Kossa‐stained slide was representative of nephrocalcinosis. We found a moderate correlation between micro‐ and macroscopic nephrocalcinosis, further supporting the techniques and quantitative methods used for classifying nephrocalcinosis severity in our study, but suggesting, as might be expected, that a single kidney tissue section gives an approximate estimate of the severity of macroscopic nephrocalcinosis assessed by 3D imaging.

Our retrospective study had some limitations. Nephrocalcinosis was identified from kidney samples collected at necropsy, and it is unknown at what point renal calcification developed in these cats. It is possible for nephrocalcinosis to have developed before CKD diagnosis. Only cats with a necropsy examination performed were included in the study. Therefore, selection bias may have occurred even though necropsy examinations were offered to all clients when their cats were euthanized at our clinics. A wide variety of maintenance diets were fed before transition to a PRD. Although the exact mineral concentrations in these diets were challenging to determine, it is reported that the median values of phosphorus content and Ca:P are 3 g/Mcal and 1.3, respectively, among 82 commercially available cat foods.^25^ Therefore, it is logical to assume that the cats that accepted a PRD after CKD diagnosis received lower dietary phosphate intake and higher dietary Ca:P than those that continued to be fed maintenance diets. Because allocation of PRD group was not randomized (all cats were offered a PRD as part of their management strategy for CKD in our study for ethical reasons), selection bias may have been introduced, even though no differences in clinicopathological variables were identified among 3 groups at baseline. Therefore, PRD effects on CKD progression and mortality should be interpreted with caution because it is possible that the unknown reason why the cats did not eat the PRD may be linked to their poorer survival. Additionally, a variety of PRDs, with differences in phosphorus content and Ca:P, were offered throughout the study period and it cannot be inferred that the results from our study can be generalized to all commercially available PRD because although the general properties of clinical renal diets are similar, variations in diet formulation exist. A prospective longitudinal study is required to further investigate the causal effect of dietary phosphate restriction on nephrocalcinosis. It is intriguing that the extent of macroscopic calcification could be evaluated using micro‐CT scanning in our study. However, the volume of FFPE kidney tissue could have been overestimated because of interference of the paraffin wax. Although measurements of multiple tissue surface areas at regular depth intervals were obtained to provide the best possible estimate of tissue volume, the VN:KT ratio potentially could have been underestimated. However, this possibility is deemed to have a relatively minor impact on the correlation between micro‐ and macroscopic nephrocalcinosis reported. Finally, nephrocalcinosis has been associated with hyperparathyroidism in human patients.^29^ This association is most likely attributable to the increased plasma and urine concentrations of calcium, urinary excretion of phosphate, and renal reabsorption of calcium stimulated by PTH, leading to renal calcification.^54^, ^55^ Fibroblast growth factor‐23 also has been suggested to be involved in nephrocalcinosis.^56^ Unfortunately, data on FGF23, PTH, and urinary electrolytes were not available in our study; and hence, their involvement in nephrocalcinosis could not be investigated. This limitation prohibits further evaluation of the potential contribution of hypercalciuria or hyperphosphaturia to nephrocalcinosis. Calcium status in the cats in our study was assessed by tCa because the biologically active iCa measurement was not available in most cats. Additional studies, including plasma FGF23, PTH, iCa, and urinary calcium and phosphate measurements, are warranted to characterize whether these factors play a more important role in nephrocalcinosis.

Collectively, we showed that higher plasma tCa concentration at CKD diagnosis and dietary phosphate restriction are independent risk factors for nephrocalcinosis although causality cannot be determined. Extraosseous calcification is a multifaceted and complex process that is actively regulated by various inducers and inhibitors.^57^, ^58^ The role of endogenous calcification inhibitors, such as magnesium, fetuin‐A, and pyrophosphate, on nephrocalcinosis remains to be explored in CKD cats. Furthermore, in cats, nephrocalcinosis did not appear to be associated with rapidity of disease progression and risk of all‐cause mortality in our study. Nonetheless, ingestion of ≥50% PRD may decrease the risk of all‐cause mortality and prolong survival in CKD cats. These apparently contradictory observations require further study. In cats, CKD is a heterogeneous syndrome with highly variable progression rates most likely driven by multiple factors. Our results implicate derangements in phosphate homeostasis in contributing to rapid progression of CKD even though nephrocalcinosis was not evident in these cats. Future prospective studies assessing the development and progression of nephrocalcinosis in cats with azotemic CKD are warranted and may lead to a new framework for diagnostic and therapeutic approaches in the management of CKD‐MBD in cats.

## CONFLICT OF INTEREST DECLARATION

Pak Kan Tang received a PhD studentship funded by Royal Canin SAS. Rosanne Jepson received funding from PetPlan, Feline Foundation for Renal Research, RVC Internal Grant, PetSavers, and consultancy agreements: Boehringer Ingelheim, Merial, CEVA. Speaking honoraria: Boehringer Ingelheim, Hills Pet Nutrition, CEVA. Rebecca Geddes received funding from Petplan, an RVC Internal Grant, The Academy of Medical Sciences and The Winn Feline Foundation; has a consultancy agreement with Boehringer Ingelheim; speaking honoraria from Boehringer Ingelheim. Jonathan Elliott received funding from Consultancies: Elanco Ltd, CEVA Animal Health Ltd, Boehringer Ingelheim Ltd, MSD Animal Health Ltd., Orion Incorp, Idexx Ltd, Nextvet Ltd, Waltham Petcare Science Institute, Kindred Biosciences Inc, Invetx Inc; grant funding from Elanco Ltd, Waltham Petcare Science Institute, Royal Canin SAS, Idexx Ltd., Zoetis Ltd, CEVA Animal Health, Member of the International Renal Interest Society.

## OFF‐LABEL ANTIMICROBIAL DECLARATION

Authors declare no off‐label use of antimicrobials.

## INSTITUTIONAL ANIMAL CARE AND USE COMMITTEE (IACUC) OR OTHER APPROVAL DECLARATION

This study was part of a larger observational cohort for which approval of the Ethics and Welfare Committee of the Royal Veterinary College was granted, URN20131258E.

## HUMAN ETHICS APPROVAL DECLARATION

Authors declare human ethics approval was not needed for this study.

## Supporting information


**Table S1** Linear mixed model analyses examining the change in clinicopathological variables over the first 365 days after a diagnosis of azotemic CKD in cats (n = 51).

**Table S2** Linear mixed model analyses examining the rate of change in CKD‐MBD variables over the first 365 days after a diagnosis of azotemic CKD in cats (n = 51).

**Table S3** Linear mixed model analyses examining the change in clinicopathological variables over the first 365 days after a diagnosis of azotemic CKD in cats (n = 51).
