# Supplementary material for: Risk factors and implications associated with renal mineralization in chronic kidney disease in cats
Source: J Vet Intern Med. 2022 Jan 19;36(2):634–46. doi: 10.1111/jvim.16363 (PMC8965253; doi:10.1111/jvim.16363)
Supplement: Supplementary file 1 — Table S1 Linear mixed model analyses examining the change in clinicopathological variables over the first 365 days after a diagnosis of azotemic CKD in cats (n = 51). [file JVIM-36-634-s003.pdf]

SUPPLEMENTARY TABLE 1. Linear mixed model analyses examining the change in clinicopathological variables over the first 365 days after a diagnosis of azotemic CKD in cats (n = 51). Summary of intercepts and the slopes of time among groups according to the severity of nephrocalcinosis (“Grade 0” vs. “Grade 1” vs. “Grade 2”).

**Variables**	**Grade 0 (n = 11)**			**Grade 1 (n = 14)**			**Grade 2 (n = 26)**		
	Intercept	Coefficient of time (month)	*P*-value	Intercept	Coefficient of time (month)	*P*-value	Intercept	Coefficient of time (month)	*P*-value
Body weight (kg)	4.3 ± 0.3	(-0.07) ± 0.03	**.01**	3.7 ± 0.2	(-0.02) ± 0.02	.24	3.9 ± 0.2	(-0.04) ± 0.02	**.01**
Albumin (g/dL)	2.98 ± 0.08	(-0.018) ± 0.013	.17	3.10 ± 0.07	(-0.004) ± 0.009	.62	3.13 ± 0.05	(-0.004) ± 0.007	.16
ALP (U/L)	79 ± 31	(-0.5) ± 3.8	.89	86 ± 27	1.9 ± 2.7	.50	61 ± 20	3.0 ± 2.1	.16
ALT (U/L)	98 ± 54	(-4.9) ± 7.9	.54	155 ± 47	(-0.2) ± 5.5	.97	95 ± 35	7.1 ± 4.3	.11
CaPP (mg^2/dL^2)	56.1 ± 5.1	2.70 ± 1.29	**.04**	44.7 ± 4.5	0.87 ± 0.89	.34	46.0 ± 3.3	0.69 ± 0.71	.34
Chloride (mEq/L)	116 ± 1	0.12 ± 0.26	.66	117 ± 1	0.01 ± 0.18	.96	118 ± 1	(-0.22) ± 0.15	.16
Creatinine (mg/dL)	2.87 ± 0.17	0.250 ± 0.074	**<.01**	2.54 ± 0.15	0.017 ± 0.056	.77	2.67 ± 0.11	0.050 ± 0.042	.24
PCV (%)	32 ± 2	(-0.5) ± 0.3	.08	35 ± 1	(-0.6) ± 0.2	**<.01**	33 ± 1	(-0.3) ± 0.2	**.04**
Phosphate (mg/dL)	5.65 ± 0.50	0.233 ± 0.115	**.05**	4.37 ± 0.44	0.089 ± 0.079	.27	4.43 ± 0.33	0.068 ± 0.064	.29
Potassium (mEq/L)	4.00 ± 0.14	(-0.017) ± 0.025	.50	4.08 ± 0.12	0.016 ± 0.017	.37	4.14 ± 0.09	(-0.005) ± 0.015	.75
Sodium (mEq/L)	150 ± 1	0.38 ± 0.22	.10	151 ± 1	0.02 ± 0.15	.88	152 ± 1	(-0.13) ± 0.13	.32
Total calcium (mg/dL)	10.00 ± 0.21	0.005 ± 0.040	.89	10.26 ± 0.18	(-0.007) ± 0.027	.79	10.37 ± 0.13	0.003 ± 0.023	.89
Total protein (g/dL)	7.7 ± 0.2	(-0.04) ± 0.03	.18	7.8 ± 0.2	(-0.01) ± 0.02	.50	7.7 ± 0.1	(-0.02) ± 0.01	.27
Urea (mg/dL)	51.4 ± 4.2	5.06 ± 1.82	**.01**	46.1 ± 3.7	1.00 ± 1.34	.46	50.5 ± 2.8	2.04 ± 1.07	.06

Outcome variables showing significant rate of change in each group ($P < .05$) are highlighted in bold. The unit used for time was month (30.4 days). Results are presented as coefficient ± standard error. Abbreviation: ALP, alkaline phosphatase; ALT, alanine aminotransferase; CaPP, calcium phosphate product.
