# Supplementary material for: Risk factors and implications associated with renal mineralization in chronic kidney disease in cats
Source: J Vet Intern Med. 2022 Jan 19;36(2):634–46. doi: 10.1111/jvim.16363 (PMC8965253; doi:10.1111/jvim.16363)
Supplement: Supplementary file 2 — Table S2 Linear mixed model analyses examining the rate of change in CKD‐MBD variables over the first 365 days after a diagnosis of azotemic CKD in cats (n = 51). [file JVIM-36-634-s002.pdf]

SUPPLEMENTARY TABLE 2. Linear mixed model analyses examining the rate of change in CKD-MBD variables over the first 365 days after a diagnosis of azotaemic CKD in cats (n = 51). Summary of *P*-values for all variables included in the model. Group represents cats in “Not eating PRD”, “Eating 10–50% PRD“ or “Eating ≥50% PRD” based on the proportion of PRD ingested during the course of CKD.

**Variables**	**Group**	**Time**	**Group*Time**
Body weight (kg)	.08	**<.01**	**.02**
Albumin (g/dL)	.48	.60	.22
ALP (U/L)	.79	.27	.52
ALT (U/L)	.59	.32	.49
CaPP (mg^2/dL^2)	.26	**.02**	.31
Chloride (mEq/L)	.68	.48	.47
Creatinine (mg/dL)	.66	**.03**	.36
PCV (%)	.87	**<.01**	.36
Phosphate (mg/dL)	.37	**.01**	.17
Potassium (mEq/L)	.34	.59	.18
Sodium (mEq/L)	.97	.71	.85
Total calcium (mg/dL)	.38	.95	.72
Total protein (g/dL)	.54	.10	.85
Urea (mg/dL)	.84	**.01**	.56

Outcome variables showing significant change over time and among groups ($P < .05$) are highlighted in bold. The unit used for time was month (30.4 days). A significant difference in the group column indicates a significant difference among the three groups at baseline for a given parameter (the start of the regression line at time 0). A significant difference in Group*Time indicates that the outcome variable differs significantly among groups (“Not eating PRD” vs. “Eating 10–50% PRD“ vs. “Eating ≥50% PRD”) over time. If Group*Time was not significant, a significant difference in Time indicates the overall rate of change of the outcome variable differs significantly from baseline over time.

Abbreviation: ALP, alkaline phosphatase; ALT, alanine aminotransferase; CaPP, calcium phosphate product.
