# Supplementary material for: Risk factors and implications associated with renal mineralization in chronic kidney disease in cats
Source: J Vet Intern Med. 2022 Jan 19;36(2):634–46. doi: 10.1111/jvim.16363 (PMC8965253; doi:10.1111/jvim.16363)
Supplement: Supplementary file 3 — Table S3 Linear mixed model analyses examining the change in clinicopathological variables over the first 365 days after a diagnosis of azotemic CKD in cats (n = 51). [file JVIM-36-634-s001.pdf]

SUPPLEMENTARY TABLE 3. Linear mixed model analyses examining the change in clinicopathological variables over the first 365 days after a diagnosis of azotemic CKD in cats (n = 51). Summary of intercepts and the slopes of time among groups (“Not eating PRD” vs. “Eating 10–50% PRD“ vs. “Eating ≥50% PRD”).

Variables	**Not eating PRD (n = 15)**			**Eating 10–50% PRD (n = 12)**			**Eating ≥50% PRD (n = 24)**		
	Intercept	Coefficient of time (month)	*P*-value	Intercept	Coefficient of time (month)	*P*-value	Intercept	Coefficient of time (month)	*P*-value
Body weight (kg)	4.4 ± 0.2	(-0.10) ± 0.02	**<.01**	3.9 ± 0.3	(-0.03) ± 0.02	.12	3.7 ± 0.2	(-0.02) ± 0.01	.12
Albumin (g/dL)	3.07 ± 0.07	(-0.017) ± 0.010	.11	3.03 ± 0.08	0.005 ± 0.010	.62	3.14 ± 0.05	0.003 ± 0.006	.60
ALP (U/L)	64 ± 26	(-1.0) ± 3.1	.76	90 ± 31	3.9 ± 3.1	.21	69 ± 20	2.5 ± 2.0	.23
ALT (U/L)	136 ± 46	(-0.7) ± 6.6	.93	140 ± 53	9.6 ± 6.5	.15	86 ± 35	1.4 ± 4.2	.74
CaPP (mg^2/dL^2)	50.6 ± 4.5	2.27 ± 1.10	**.05**	40.2 ± 5.2	1.55 ± 1.09	.16	49.5 ± 3.4	0.35 ± 0.71	.62
Chloride (mEq/L)	117 ± 1	(-0.3) ± 0.2	.20	118 ± 1	0.1 ± 0.2	.66	117 ± 1	0.1 ± 0.1	.66
Creatinine (mg/dL)	2.77 ± 0.15	0.156 ± 0.067	**.02**	2.56 ± 0.17	0.026 ± 0.067	.70	2.68 ± 0.11	0.060 ± 0.044	.18
PCV (%)	34 ± 1	(-0.7) ± 0.2	**<.01**	33 ± 2	(-0.2) ± 0.2	.33	33 ± 1	(-0.4) ± 0.1	**.01**
Phosphate (mg/dL)	4.99 ± 0.46	0.246 ± 0.096	**.02**	4.05 ± 0.52	0.122 ± 0.094	.21	4.76 ± 0.34	0.029 ± 0.061	.63
Potassium (mEq/L)	4.19 ± 0.12	(-0.031) ± 0.021	.15	3.92 ± 0.14	(-0.004) ± 0.023	.86	4.10 ± 0.09	0.017 ± 0.014	.24
Sodium (mEq/L)	151.5 ± 0.7	0.002 ± 0.177	.99	151.2 ± 0.8	0.115 ± 0.191	.55	151.3 ± 0.5	(-0.010) ± 0.117	.93
Total calcium (mg/dL)	10.11 ± 0.18	(-0.016) ± 0.033	.63	10.14 ± 0.21	0.022 ± 0.034	.53	10.39 ± 0.14	(-0.002) ± 0.021	.92
Total protein (g/dL)	7.9 ± 0.1	(-0.03) ± 0.02	.21	7.6 ± 0.2	(-0.02) ± 0.02	.50	7.7 ± 0.1	(-0.01) ± 0.01	.32
Urea (mg/dL)	48.5 ± 3.7	3.47 ± 1.60	**.04**	48.1 ± 4.2	0.94 ± 1.68	.58	50.7 ± 2.8	2.25 ± 1.06	**.04**

Outcome variables showing significant rate of change in each group ($P < .05$) are highlighted in bold. The unit used for time was month (30.4 days). Results are presented as coefficient ± standard error.

Abbreviation: ALP, alkaline phosphatase; ALT, alanine aminotransferase; CaPP, calcium phosphate product.
